# The effects of a culturally-tailored campaign to increase blood donation knowledge, attitudes and intentions among African migrants in two Australian States: Victoria and South Australia

**DOI:** 10.1371/journal.pone.0188765

**Published:** 2017-11-30

**Authors:** Kate L. Francis, Michael J. Polonsky, Sandra C. Jones, Andre M. N. Renzaho

**Affiliations:** 1 Centre for Health and Social Research, Australian Catholic University, Melbourne, Australia; 2 Department of Marketing, Deakin Business School, Deakin University, Burwood, Australia; 3 School of Social Sciences and Psychology, Western Sydney University, Penrith, Australia; Queensland University of Technology, AUSTRALIA

## Abstract

Research suggests that African migrants are often positively predisposed towards blood donation, but are under-represented in participation. A culturally-tailored intervention targeting the African migrant community in Australia was developed and implemented, to enhance knowledge about blood donation, improve attitudes towards donating, increase intentions to donate blood, and increase the number of new African donors in Australia. Four weeks after a targeted campaign, a survey evaluation process commenced, administered face-to-face by bilingual interviewers from the African community in Melbourne and Adelaide, Australia (community survey). The questionnaires covered demographics, campaign awareness, blood donation knowledge and intentions, medical mistrust and perceived discrimination, and were analysed to evaluate changes in knowledge and intention. Sixty-two percent of survey participants (n = 454) reported being aware of the campaign. With increasing campaign awareness, there was a 0.28 increase in knowledge score (p = .005); previous blood donation was also associated with an increased blood donation knowledge score. Blood donation intention scores were not associated with campaign awareness (p = 0.272), but were associated with previous blood donation behaviour and a positive blood donation attitude score. More positive scores on the blood donation attitude measure were associated with increasing blood donation intentions, self-efficacy and campaign awareness (score increases of 0.27, 0.30 and 0.04, respectively, all p<0.05). Data were collected on the ethnicity of new blood donors in six blood collection centres before and after the intervention, and independent of the intervention evaluation survey. These data were also used to assess behavioural changes and the proportions of donors from different countries before and after the survey. There was no difference in the number of new African migrant donors, before and after the intervention. The culturally-relevant marketing campaign was associated with improved blood donation knowledge and attitudes, but there was no short-term change in blood donation intentions or the number of African donors.

## Introduction

Encouraging blood donation by all members of the community is important for the operation of effective health systems. Research from around the world has found that minority groups and migrants are frequently under-represented in blood donation participation [1–6]. For example, based on the UK [4] census and National Health Service data, it has been reported that there are vast differences in ethnic group donation participation: 22.1 donors per 1000 population for the white British population, 1.84 donors of African descent, and 1.59 among those of Asian Bangladeshi descent. Similar discrepancies have been reported in Atlanta, USA [7], with 11 donors per 1000 for Whites, 6 donors per 1000 for African Americans, and 3 donors per 1000 for Hispanics. A comparison of population data with 2010 donor data in the USA [8] indicated that the African American community comprised 12.6% of the population, whereas only 4.9% of blood donors were members of that community. Large differences in donation rates have also been found in Germany, with 21.1% of non-migrants donating blood compared to 11.4% of migrants [1].

Globally, facilitating donations from migrants and minority communities is important, especially if matched blood types are required to prevent transfusion complications [9–11]. Therefore, it is important to design and implement strategies that facilitate blood donation among migrant and ethnic communities [12, 13], and to overcome the barriers and enhance the enablers to participation through targeted interventions. A review of the literature on the barriers and motivations to voluntarily giving blood in Sub-Saharan Africa [14] identified that many of these barriers and motivators also exist in developing countries (e.g., lack of knowledge, fear, and lack of information), which is consistent with other reviews of blood donation barriers focusing on non-migrant communities [15].

Research outside Australia has found that knowledge about blood donation plays a significant role in intentions to donate blood generally [16, 17], and is also important for migrants [12]. Inaccurate knowledge about blood donation could, therefore, serve as a barrier to whether or not potential African donors (or others), in fact, donate blood in Australia. An example of an important barrier that migrants face is their possible lack of knowledge and understanding of host country blood donation procedures, which may be substantially different from those in their home country. For example, in Africa, blood services often rely on direct replacement donation (i.e., recipients and their families seek to source blood donations from known individuals) [18], rather than from a centralized anonymously donated blood supply [14, 19]. Differences in donation processes between countries create additional pressure to provide effective communication to explain the differences which, therefore, requires appropriate communication adaptation. Research has suggested that the failure to adapt health messages is another impediment for migrant participation in health encounters [1, 6], such as blood donation [6].

Another barrier for migrant blood donation participation is the issue of trust in the health system. For example, research in the USA and Canada has found that minority donors distrust biomedical organisations because of negative historical events, which creates barriers to participation in blood donation [5, 6, 20, 21]. While earlier research suggests that medical mistrust was not a blood donation barrier for African migrants in Australia, these same migrants believed that their blood would not be wanted by the host population, based on the migrants’ perceived discrimination in the wider community [22, 23]. The role of perceived discrimination as a barrier to blood donation has also been identified by African migrants in Canada [5] and France [12]. Perceived discrimination was also found to impede African Americans’ intentions to donate blood [11], as well as impede blood donation by African migrants in Australia [23]. Thus, perceived discrimination also needs to be considered when evaluating the blood donation intentions of migrants.

Participating in the blood donation process is also a way to bring about enhanced social inclusion in under-represented groups [5, 12]. Facilitating social inclusion of migrants in host countries, through health services such as blood donation, is of growing importance given the increasing global influx of migrants into developed countries [24]. New migrant donors are indeed important, however, migrants are not always easily integrated into host countries. As a result, long-lasting negative effects on inclusion [25] and a negative influence on migrants’ participation in health activities [26], including blood donation behaviour [5], can occur.

In Australia the African community is growing rapidly, and, according to the 2011 Australian census, African migrants represent 1.8% of people aged 18–75 years, with 47.0% of this population having arrived since 2001 [27]. While this community is positively predisposed towards blood donation [28], the rates of migrant donation in Australia appear to be lower than in the general community, for example, 2.4% in a sample of 425 respondents compared to 3.5% in the general community [29]. This positive predisposition towards donation will not only help ensure a sustainable blood supply, but assists in facilitating the social inclusion of African migrants in Australia [23].

Given the purported benefits of targeted, culturally-sensitive interventions [1, 12, 13], this research compares the impact on those with an awareness of a social marketing campaign with those who were not aware of the campaign. It also investigates campaign awareness on African migrants’ behaviours towards blood donation, as well as their intentions to donate, attitudes towards donating blood and their knowledge about blood donation in Australia.

## Materials and methods

### Development and implementation of the intervention

Research suggests that health interventions which express the voice and experience of those being targeted are more effective in changing attitudes and behaviours [13, 30, 31]. As a result, a blood donation communication campaign was developed in conjunction with African migrants, ensuring that the communication was salient to the targeted community and, thus, more likely to engender changes in knowledge, intentions and behaviours [13, 30, 31]. The materials were based on earlier quantitative and qualitative research in the African community undertaken in 2010 [22, 23, 28, 29, 32, 33]. Seven additional focus groups were conducted with African migrants in 2013/2014, in a location different from that for the intervention. The focus groups built on the earlier research and extended discussions on blood donation, and possible alternative messages and formats for the messages.

The outcome of the focus groups was a communication campaign entitled “Blood from Everyone for Everyone”. The associated materials included: a booklet (available in hard copy, online and on USB); four different posters; and a short video (available online and on USB). All materials and voice-overs of videos incorporated members of the African community engaged in the blood donation process. Materials were made available in English and in Arabic, Kirundi and Swahili to overcome any English literacy issues. French was not used in the intervention as, within Australia, the migrants from Eastern Africa, including the Great Lakes and Western Africa, have been found to speak Swahili and Arabic predominantly, with most of those from Western Africa speaking Broken English [34]. The straightforward and visual nature of the information provided, combined with extensive pre-testing with members of the target communities, ensured that those engaging with the materials would have been able to read/watch and understand the materials in one of the four languages.

Two separate parts to the assessment of the intervention are reported in this paper. The first was the community survey that was started four weeks after the materials were distributed. The second assessment was a donor centre survey that took place at blood collection centres one year prior to the intervention, and again after the intervention, via email (as directed by the Australian Red Cross Blood Service).

### Community survey

Bilingual workers were recruited and trained to assist in the distribution of the intervention materials in the African community, as well as to undertake the face-to-face participant interviews. Materials were distributed in August 2015 through local religious institutions (churches, mosques etc.), restaurants, cafés and community groups in metropolitan Melbourne and Adelaide, which are home to 27.0% of the African migrant community in Australia.

Interviewer-administered surveys were used to assess the impact and reach of the intervention materials, and commenced four weeks after the initial material dissemination. Trained bilingual workers used their own community networks to access potential participants, and a snowball approach was used to recruit additional participants. Throughout the data collection, the country of birth data were monitored based on the United Nations’ geo-scheme for Africa to ensure all regions (Central—9 countries, Eastern—20 countries, Northern -7 countries, Southern—5 countries, Western—17 countries) were represented. In Australia, 74% of the African migrants come from Southern and Eastern Africa [35] and, thus, would be expected to comprise the majority of the sample. The respondents’ home country regions were broadly consistent with the composition of African migrants in Australia (adjusted to remove the white South African migrant community).

Eligibility criteria required that the participants had to have been born in Africa, were over 18 years old, lived in metropolitan Adelaide or Melbourne, and were not short-term visitors. In addition, no more than two participants from the same household could complete the survey. A $15 gift card was given to each participant to thank them for their time. Ethics approval was granted from the Australian Red Cross Blood Service Human Research Ethics Committee, reference number: 2014#24, as well as from each researcher’s university ethics committee, as follows: The Australian Catholic University Human Research Ethics Committee Register No: 201500012R; the University of Western Sydney Human Research Ethics Committee: H10963; and the Deakin University Human Research Ethics Committee: 2015–001).

The interview questions were read to the participant by the interviewer in the language that was most appropriate to the respondent. An electronic tablet used the Qualtrics software system to enter data and minimize transcription errors. The data file from Qualtrics was exported to Stata for analysis.

### Donor centre survey

It was necessary to identify the composition of donors’ pre and post intervention, as the Blood Service does not include this data in their donor database. To do this, the country of birth donor data were collected from all donors at four blood collection centres in Melbourne, and two centres in Adelaide, in July and August 2014, that is, 12 months before the intervention. The donor centres were selected based on a review of Australian census data that identified which centres were located in areas with the highest numbers of residents of African descent. Potential donors (i.e., actual donors and those who were deferred) attending the targeted collection centres were asked to complete a short survey which only asked them about their country of birth and their parents’ country of birth. Based on Blood Service data, there were 9850 donor visits to the centres during this time, and 3445 (35.0%) donors completed the survey. The response rate for new donors on the country of birth question in the six collection centres was 14.5%, or 134 of the 925 new donors.

Post-intervention, country of birth donor data were collected using an online survey. The survey link was emailed by the Blood Service to all new donors who attended the six targeted collection centres between November and December 2015. New donors were only asked about their country of birth and their parents’ country of birth, thus ensuring the survey was independent of the community survey assessing the intervention (described below). Based on Blood Service data, 29.4% of all new donors at the six centres completed the post-intervention country of birth questionnaire.

## Participants

### Community survey

A total of 473 interviews were conducted with African migrants in the wider community: 68.0% in Victoria (n = 323), and 32.0% in South Australia (n = 150). Nineteen were excluded as the respondent was not born in an African country, leaving a usable sample of 454. The participants in the sample had a mean age of 33 years (median 32, range: 18–67), 52.0% were female, and the mean duration of residence in Australia was 9.1 years. A quarter of participants (n = 115) reported that they had previously donated blood; 27.8% of those who had donated blood (n = 32) had done so in Australia (i.e., 7.0% of the total sample). The composition of the community, post-intervention survey is described below.

### Donor centre survey

The pre-intervention donor centre survey on country of birth was completed by n = 134 new donors (57.0% female), and the post-intervention survey on country of birth was completed by 394 new donors (60.0% female). This survey did not include any attitudinal measures and was solely designed to evaluate changes in the demographic composition of new donors, thus, the two surveys are independent. The small number of respondents of African descent identified in the new donor surveys means it is unlikely that these people would have been involved in the community survey.

### Measures

#### Community survey

The community survey was designed to assess the awareness and attitudinal impacts of the intervention. It contained a number of demographic questions, and items on blood donation knowledge, attitudes, behaviour, and campaign awareness. Given that previous research findings identified perceived discrimination [5, 12, 22, 23] and medical mistrust [5, 6, 20, 21, 23] as barriers specific to African minority and ethnic group participation in blood donation, these factors were also included in the survey to allow us to account for their impact on the outcome measures of blood donation intentions and knowledge. As blood donation attitudes have been found to predict blood donation intentions [36], a measure to assess attitudes was also included.

#### Demographic questions

Data were collected on respondents’ age, education level, employment status, religion, country of birth, time lived in Australia, and past blood donation behaviour.

#### Campaign awareness

The interviewer showed participants each of the four intervention resources and asked whether they had seen each item. The overall exposure variable was based on participants indicating they had seen the book/brochure, poster, website or video. Responses could range from 0 (not seen any materials) to 4 (seen all materials). The USB was not included in this calculation, as receiving it would not necessarily mean they had plugged it in and watched or read the campaign materials. Unfortunately, there is no way of knowing whether respondents actually read the booklet or watched the video in full, thus, exposure may not reflect the depth of engagement with materials. This issue is a problem with many assessments of material engagement in health promotions.

#### Blood donation knowledge

The blood donation knowledge questionnaire developed by Renzaho and Polonsky [28] consists of 16 true/false questions. Internal consistency for the dichotomous scale was assessed by the Kuder–Richardson-20 method (KR20 = 0.625).

#### Blood donation intentions

Three items assessing blood donation intentions were summed and averaged; responses could range from 1 to 5, with low scores indicating low intention to donate blood. These items were the same as those used in a previous study with this community [32]. Cronbach’s alpha of internal consistency was 0.965 for this scale.

#### Blood donation attitudes

Four items assessing blood donation attitudes were summed and averaged; responses could range from 1 to 5, with low scores indicating a negative attitude toward blood donation. These items were the same as those used in a previous study with this community [32]. Cronbach’s alpha of internal consistency was 0.934 for this scale.

#### Self-efficacy

Four items assessing blood donation self-efficacy were summed and averaged; responses could range from 1 to 5, with low scores indicating low self-efficacy toward blood donation. These items were the same as those used in a previous study with this community [32]. Cronbach’s alpha of internal consistency was 0.928 for this scale.

#### Perceived discrimination

Respondents’ perceptions of discrimination in Australia were assessed using the perceived discrimination scale from a previous study [33]. The scale addresses three types of discrimination—personal (4 items), fitting in (3 items) and societal (3 items); responses are measured on a 5-point Likert scale, ranging from ‘strongly disagree’ to ‘strongly agree’. Item scores were combined to give one single overall score for each type of discrimination. Higher scores indicate a greater perception of the form of discrimination. Cronbach’s alpha internal consistency scores were 0.924, 0.800 and 0.926 for the personal, fitting in and societal discrimination scales, respectively.

#### Medical mistrust

Respondents’ general views of the medical system were assessed using a seven-item medical mistrust scale developed in a previous study [33]. Responses are on a 5-point Likert scale, ranging from ‘strongly disagree’ to ‘strongly agree’. A mean score was computed for this variable, with higher scores indicating more mistrust. Cronbach’s alpha of internal consistency was 0.901 for this scale.

### Donor centre survey

New donors attending the targeted centres answered a questionnaire on their country of birth, which was administered before and after the intervention, independently of the community survey. The data come from four blood collection centres in Melbourne and two centres in Adelaide. The pre-intervention data were collected in July and August 2014, and follow-up data via an electronic survey in November and December 2015. The country of birth data included all new donors (successful donations and deferrals) who had an appointment. The data were anonymous and, therefore, cannot be related to responses within the community survey.

## Data analysis

### Community survey

There were two components of the data analysis. First, whether there were demographic differences between those who were aware of the campaign materials and those who were not. Second, a regression analysis was carried out to determine what variables (socio-demographic factors, attitudes, medical mistrust and discrimination) were associated with the outcome measures of blood donation knowledge, attitudes and donation intentions.

### Donor centre survey

Behavioural data were analysed using Chi-squared tests to compare the number of new donors from African countries before and after the intervention.

## Results

### Community survey

The first issue was whether the respondent had seen the materials. As this is a self-reported measure, it is possible that people who have ‘seen items’ did not, in fact, review them in detail. For example, the web metrics reported overall time on the intervention web site, but not whether people who viewed the online videos, watched the whole video. Overall, 61.8% (n = 281) of respondents reported seeing at least one piece of campaign material (1 item—30.4%; 2 items—18.9%; 3 items—7.0%; and 4 items—5.5%). Table 1 reports the differences in demographics and attitudinal variables between those who had seen materials and those who had not.

**Table 1:** Comparison of those who had seen campaign materials and those who had not.

	Total sample n (%)	Seen materials n (%)	Not seen materials n (%)
**Gender**			
Male	216 (47.6)	143 (50.9)	73 (42.2)
Female	238 (52.4)	138 (49.1)	100 (57.8)
**Age (in years), n = 472**			
Mean (sd)	33.4 (9.3)	32.9 (9.0)	34.3 (9.7)
Median	32	32	33
Range	18–67	18–67	18–65
**Religion**			
Christian	339 (74.7)	211 (75.1)	128 (74.0)
Islam	60 (13.2)	38 (13.5)	22(12.7)
None/Prefer not to say/Other	55 (12.1)	32 (11.4)	23 (13.3)
**Education**			
Primary, Secondary or Other	102 (22.5)	68 (24.2)	34(19.7)
University or TAFE	352 (77.5)	213 (75.8)	139 (80.3)
**Employment**			
Not employed	107 (23.6)	73 (26.0)	34 (19.7)
Employed full/part-time/casual	340 (74.9)	204 (72.6)	136(78.6)
Other	7 (1.5)	4 (1.4)	3 (1.7)
**Prior to migration, lived in:**			
Large city	227 (50.1)	139 (49.6)	88 (50.9)
Town or suburban area	138 (30.5)	91 (32.5)	47 (27.2)
Rural area/village	88 (19.4)	50 (17.9)	38 (22.0)
**Years lived in Australia**^a^ **(n = 459)**			
Mean (sd)	9.1 (5.7)	8.6 (4.9)	9.9 (6.8)
Median (years)	9	9	9
Range (years)	0–34	0–24	0–34
**Country of birth: African Region**^a^			
Central	34 (7.5)	22 (7.8)	12 (6.9)
Eastern Africa	208 (45.8)	115 (40.9)	93 (53.8)
Northern Africa	92 (20.3)	67 (23.8)	25 (14.5)
Western Africa	89 (19.6)	53 (18.9)	36 (20.8)
Southern Africa	31 (6.8)	24 (8.5)	7 (4.0)
**Blood donor**^a^			
No	339 (74.7)	199 (70.8)	140 (80.9)
Yes	115 (25.3)	82 (29.1)	33 (19.1)

^**a**^Indicates a significant difference between groups p<0.05

Those who had seen the materials had lived in Australia for less time (8.6 years) than those who had not seen the materials (9.9 years) (F = 16.43, p = 0.033), which may reflect a desire by newer migrants to learn more about Australian practices. Those who had seen the materials were more likely to have donated blood previously (29.0% versus 19.0%; X^2^ = 5.78, p = 0.016), which might suggest they were more engaged or interested in blood donation. With regard to region of birth, those from Northern African countries were more likely to have seen the materials, and those from Eastern Africa were less likely to have seen the materials (X^2^ = 12.0, p = 0.017).

Multiple linear regression analysis, using Stata version 14.2, was then performed with three outcome variables (blood donation knowledge, attitudes towards blood donation and blood donation intentions) as the dependent variables. The primary goal of the intervention was to increase donation intentions, however, the transtheoretical model of health behaviour suggests that people advance through five stages—pre-contemplation, contemplation, preparation, action, and maintenance [37]. The final stage is to undertake the behaviour, but people need to move through this decision process to get to that final point, therefore, health interventions, including those for blood donation, need to advance target audiences along the process [38]. A lack of blood donation knowledge has been identified as critical, impeding intentions and donations, as are attitudes towards blood donation [39]. Thus, improving knowledge and/or attitudes are critical for improving blood donation intentions [23] or moving migrants through the transtheoretical model of health beliefs. The first regression examines whether the intervention enhances blood donation knowledge.

The regression model for blood donation knowledge (Table 2) explained 22.7% of the variance (F (28,399) = 5.49, p < .001). For each unit increase in exposure to materials, we expect a 0.28 unit increase in knowledge score (p = .005), while holding other variables constant. Previous blood donation was associated with knowledge, with an expected increase in knowledge score of 0.72 (p = .018) for those who have donated outside of Australia, and 1.4 for those who had donated blood in Australia (p = .001) holding other variables constant. Higher knowledge scores are predicted for those not categorised as unemployed, with those employed expected to have a 1.20 unit greater score on knowledge (p < .001); and those categorized as “other” had a 1.95 unit increase in their knowledge scores (p = .040) when all other variables were held constant. Two variables were negatively associated with blood donation knowledge scores: the first being from the Western region of Africa compared to the Central region (-1.17 unit lower score on knowledge, p = .014), and the second being from a rural area pre-migration compared with a large city (-.68 unit lower score on knowledge, p = .027) when all other variables were held constant.

**Table 2:** Regressions of intentions attitudes and knowledge.

	Intentions				Attitudes				Knowledge			
	Coeff.	P	95% CI		Coeff.	P	95% CI		Coeff.	p	95% CI	
			Lower	Upper			Lower	Upper			Lower	Upper
**Seen materials**	0.020	0.272	-0.016	0.057	0.046	**0.044**	0.001	0.091	0.282	**0.005**	0.086	0.478
**Outcome measures**												
Intentions					0.269	**0.000**	0.150	0.388	0.446	0.101	-0.087	0.980
Knowledge	0.015	0.101	-0.003	0.033	0.019	0.099	-0.004	0.041				
Attitudes	0.176	**0.000**	0.098	0.254					0.363	0.099	-0.069	0.795
Self-efficacy	0.740	**0.000**	0.675	0.805	0.302	**0.000**	0.186	0.419	-0.420	0.120	-0.950	0.109
Medical mistrust	0.036	0.185	-0.017	0.089	0.033	0.326	-0.033	0.099	0.173	0.244	-0.118	0.464
Personal discrimination	-0.039	0.235	-0.103	0.025	-0.062	0.123	-0.142	0.017	-0.067	0.706	-0.418	0.283
Not fitting in discrimination	0.024	0.389	-0.031	0.080	0.057	0.105	-0.012	0.125	0.080	0.602	-0.222	0.383
Societal discrimination	-0.009	0.734	-0.062	0.044	0.033	0.317	-0.032	0.099	0.217	0.138	-0.070	0.504
**Age**	0.001	0.816	-0.004	0.005	0.001	0.832	-0.005	0.006	-0.023	0.072	-0.048	0.002
**Years lived in Australia**	-0.008	0.052	-0.015	0.000	0.004	0.441	-0.006	0.013	-0.017	0.432	-0.058	0.025
**Gender**												
Male	Ref				ref				ref			
Female	0.030	0.471	-0.051	0.110	0.060	0.241	-0.040	0.159	0.158	0.480	-0.281	0.597
**Education**												
Primary school	ref				ref				ref			
Completed secondary	-0.097	0.475	-0.363	0.169	0.337	**0.043**	0.010	0.664	0.109	0.882	-1.338	1.556
Completed TAFE	-0.042	0.757	-0.307	0.223	0.417	**0.012**	0.092	0.742	-0.569	0.437	-2.010	0.871
Completed university	-0.062	0.643	-0.327	0.202	0.396	**0.017**	0.071	0.721	0.569	0.437	-0.870	2.008
Completed postgrad	-0.060	0.664	-0.330	0.210	0.397	**0.019**	0.066	0.729	0.777	0.298	-0.690	2.244
Other	0.080	0.703	-0.330	0.490	0.279	0.280	-0.228	0.785	-0.066	0.954	-2.297	2.166
**Employment**												
Not employed	ref				ref				ref			
Employed full/part-time/casual	-0.050	0.346	-0.154	0.054	-0.058	0.376	-0.186	0.071	1.214	**0.000**	0.661	1.767
Other	0.233	0.185	-0.112	0.578	-0.279	0.199	-0.705	0.147	1.958	**0.040**	0.088	3.828
**Country of birth: African Region**												
Central	ref				ref				ref			
Eastern Africa	-0.030	0.713	-0.187	0.128	0.097	0.325	-0.097	0.292	-0.534	0.220	-1.388	0.321
Northern Africa	0.103	0.241	-0.070	0.276	0.157	0.151	-0.057	0.370	-0.931	0.052	-1.869	0.008
Western Africa	0.010	0.915	-0.165	0.184	0.251	0.021	0.038	0.465	-1.175	**0.014**	-2.114	-0.235
Southern Africa	-0.283	**0.010**	-0.499	-0.067	0.084	0.540	-0.185	0.353	-0.814	0.176	-1.994	0.367
**Religion**												
None/Prefer not to say/Other	ref				ref				ref			
Christian	-0.157	**0.015**	-0.284	-0.031	0.264	**0.001**	0.109	0.420	-0.027	0.939	-0.719	0.666
Islam	-0.091	0.262	-0.249	0.068	0.205	**0.040**	0.010	0.401	-0.574	0.192	-1.438	0.289
**Prior to migration, lived in…**												
Large city	ref				ref				ref			
Town or suburban area	-0.008	0.856	-0.101	0.084	0.014	0.811	-0.100	0.128	0.163	0.522	-0.337	0.663
Rural area/village	-0.062	0.274	-0.175	0.050	0.040	0.573	-0.099	0.179	-0.685	**0.027**	-1.292	-0.078
**Blood donor**												
No	ref				ref				ref			
Yes—not in Australia	0.132	0.112	-0.031	0.295	-0.057	0.577	-0.259	0.144	1.428	**0.001**	0.551	2.305
Yes—in Australia	-0.022	0.700	-0.132	0.089	-0.144	**0.038**	-0.280	-0.008	0.722	**0.018**	0.125	1.320
Constant	0.172	0.527	-0.362	0.705	0.530	0.114	-0.128	1.187	8.963	0.000	6.198	11.728

The regression model for blood donation attitudes (Table 2) explained 52.6% of the variance (F (28,399) = 17.95, p < .001). For each unit increase in exposure to materials, we expect a 0.05 unit increase in attitude score (p = .044), while holding other variables constant. Blood donation intentions and self-efficacy were associated with higher blood donation attitude scores (0.26 unit greater score on attitude for each unit increase on intentions (p < .001), and 0.30 unit greater score on attitudes for each unit increase on self-efficacy (p < .001). Completion of secondary school, TAFE or vocational course or any university education compared with only primary school education were all associated with increased attitude scores. Identifying as religious was associated with a higher attitude score (0.26 unit increase for Christians (p = 0.001), and 0.21 unit increase for those of the Islamic faith (p = 0.040)) compared with no/other religion. Being from the Western region of Africa compared to the Central region was associated with higher attitude score (0.25 units, p = .021). Having donated blood outside of Australia was associated with a lower score on blood donation attitude compared with having no donation history (-0.14 unit decrease, p = 0.038).

The regression model for blood donation intentions (Table 2) explained 78.2% of the variance (F (28,399) = 55.78, p < .001). Having seen the intervention materials was not statistically-significantly related to the respondent’s blood donation intention score (p = .272). Statistically-significant predictors of blood donation intentions were: blood donation attitude (0.74 unit greater score on intentions for each unit increase on attitudes (p < .001)), and blood donation self-efficacy (0.18 unit greater score on intentions for each unit increase on self-efficacy (p < .001)). Two variables were negatively associated with blood donation intention scores: the first being Christian compared to having no religion (-0.16 unit decrease, p = .015), and being born in Southern Africa as opposed to Central (-0.28, unit decrease p = .010).

### Donor centre survey

The data on new donor country of birth (Table 3) shows no statistically-significant differences between the composition of new donors, before and after the intervention, as in all cases zero was contained in the 95% confidence interval. While the focus of the intervention was on African migrants, we assessed the effects across a range of migrant classifications to determine whether there were other unintended effects, and none arose. Given that there was no decrease in the portion of Australian-born donors before and after the intervention, it can be concluded that there was no impact across the aggregate group of non-Australian donors.

**Table 3:** Country of birth for new donors pre- and post-intervention.

Country of birth	Pre-Intervention n (%)	Post- Intervention n (%)	Difference	95% CI difference		*χ*^2^	p
				Lower	Upper		
African/Middle Eastern	3 (2.23%)	9 (2.28%)	0.05	-2.80	3.00	0.00	0.976
Asian	17(12.67%)	73 (18.53%)	5.84	-1.00	12.70	2.41	0.120
Pacific (other than Australia)	6 (4.48%)	8 (2.03%)	-2.44	-6.20	1.30	2.32	0.128
Australian	97 (72.39%)	282 (71.6%)	-0.81	-9.50	7.90	0.03	0.856
European	8 (5.97%)	12 (3.05%)	-2.92	-7.20	1.40	2.35	0.126
North American	2 (1.49%)	4 (1.02%)	-0.48	-2.70	1.80	0.20	0.652
South American	1 (0.75%)	3 (0.76%)	0.02	-1.60	1.70	0.00	0.986
Not specified overseas		3 (0.76%)					
**Total**	134 (100%)	394 (100%)					

## Discussion

The results of the evaluation indicate that African migrants who were aware of the targeted campaign had greater blood donation knowledge, as well as more positive attitudes towards blood donation. However, campaign awareness was not associated with improved intentions to donate, nor did donation participation by African migrants increase after the intervention. It may be that the results suggest that the enhancement of knowledge and improvements in attitudes will move the targeted community through the various stages of the transtheoretical model, but participants had not yet advanced to the action stage. Other health promotion research examining blood donation suggests that sustained activities need to be undertaken to move people through the various stages of this model [40]. Regression analysis is not causal, therefore, perhaps those with higher blood donation knowledge or more positive blood donation attitudes are more likely to notice a campaign to enhance blood donation participation.

We did also seek to consider the role of covariate variables. We did, however, find some relationships were important but were inconsistent across regression models. Self-efficacy and ‘being Christian’ were found to be two significant models which increased intentions and attitudes. In the case of self-efficacy, it makes sense that people who believe they can act are more likely to intend to give and are also more positive in regard to the act they are able to undertake [41]. It is unclear as to why the role of religion would have an impact, other than the fact that in some religions there is confusion in migrant communities regarding the acceptability of donating blood [12].

We also found that ‘having donated blood previously in Australia’ was significant in increasing attitudes and knowledge. This, again, makes sense, as people who have given blood previously in Australia would have a greater understanding of the process and, thus, more blood donation knowledge, as well as be more likely to have positive blood donation attitudes given they have engaged in the action [39]. It is, however, surprising that this also did not translate into greater intentions to donate, as past donors are generally more likely to give in the future than those who have not donated [42].

The lack of any apparent impact of the intervention on intentions is concerning, as research in the USA [13] and France [12] has found that ethnically-targeted interventions can increase donation intentions and behaviour. It does need to be highlighted that the behavioural measures in this study only focused on new donors and, thus, there may have been increases in the donation activities of those who had already donated. Further, it was not possible to assess in the interview how well participants had engaged with the materials (e.g., did they read all the booklet or watch the whole video?). Previous donors may have been more likely to recall seeing information that relates to a behaviour they already engaged in, hence the large percentage (7%) who reported previously donating blood. Those who have already donated may also be more interested in blood donation. Research suggests topic interest increases survey participation [43].

Furthermore, we could not assess the material distribution impact in a formal sense, other than asking who was aware of the materials. Although, pleasingly, the reach appeared quite high (62%), greater dissemination or more intensive repetition may have been required to bring about community level change. Although we believe the dissemination of materials by community members is a realistic way to promote campaigns (with limited spend), we acknowledge future campaigns may benefit from a more intensive communication strategy.

It is also possible that the proximity of the evaluation to the intervention may explain the lack of significant results. The impact of the campaign was assessed starting four weeks after the intervention, which may not have been sufficient time for increases in knowledge to lead to improvements in attitudes and intentions. Given that lack of blood donation knowledge has been identified as an important barrier to blood donation [15], including among individuals from developing countries [39], and African migrants specifically [28], it is possible that the intervention achieved changes in knowledge (an antecedent to changing behaviour), but that the flow-on effects were not captured in the short-term evaluation, as it takes time to move through the transtheoretical model.

In addition, the lack of behavioural change may be related to the very small sample of non-Australian new donors. This finding is consistent with research which has suggested donation rates in Australia across all communities are relatively low [2], and that the lack of ethnic and migrant inclusion is a global issue [1]. The combination of a small sample frame (non-Australian new donors) and the potentially higher barriers to survey completion of this group of donors attending centres, means that the sample size was too small for analysis of changes in the targeted group. Future studies that aim to increase blood donation participation in specific migrant groups would benefit from having country of birth data routinely collected and reported by Blood Service donor management systems, thus addressing the issue of low response rates to surveys. This limitation arose from having to conduct the post-intervention country of birth survey online as it was only available in English, possibly limiting the ability of people from migrant backgrounds to respond. Communication with staff at blood donation collection centres identified there were a few instances where translators were used, as required if a person is not sufficiently literate in English. At the time of this study, Australia was one of many countries that did not collect data on donors’ country of birth [6]. However, the collection of such data would allow for broader tracking of behaviours in migrant and ethnic communities, to better identify the efficacy of targeted and non-targeted interventions.

Having culturally-targeted campaigns does have benefits, as campaign awareness was associated with increased knowledge and attitudes (generally argued as a precursor of intentions and behaviours). The role of such initiatives with various migrant groups needs to be further explored. It may be that a multi-faceted approach is required whereby informational communications are followed up with other types of interventions—such as targeted community collections—to address other issues associated with donation barriers. It may also be useful for blood services to work with other governmental agencies, given the broader benefits of blood donation such as facilitating social inclusion [22, 23]. Such activities would make blood donation more salient, addressing the critical issue of maintaining adequate blood supplies, enabling appropriate health services to be provided, and enhancing migrants’ social inclusion.
